# Nitrogen loading effects on nitrification and denitrification with functional gene quantity/transcription analysis in biochar packed reactors at 5 °C

**DOI:** 10.1038/s41598-018-28305-0

**Published:** 2018-06-29

**Authors:** Su He, Lili Ding, Yao Pan, Haidong Hu, Lin Ye, Hongqiang Ren

**Affiliations:** State Key Laboratory of Pollution Control and Resource Reuse, School of the Environment, Nanjing University, Nanjing, 210023 Jiangsu PR China

## Abstract

This study investigated the nitrogen transformation rates of different nitrogen-loading (20, 30, and 50 mg TN/L) biochar packed reactors (C:N:P = 100:5:1) within 125 days at 5 °C. The results showed that high nitrogen loading resulted in an NH_4_^+^ (TN) removal efficiency decline from 98% (57%) to 83% (29%), with biochar yielding a higher NH_4_^+^, TN and DON removal rate than conventional activated sludge. Moreover, all biochar packed reactors realized a quick start-up by dropping in temperature stage by stage, and the effluent dissolved organic nitrogen (DON) concentrations of R_20_, R_30_, and R_50_ were 0.44 ± 0.18, 0.85 ± 0.35, and 0.66 ± 0.26 mg/L, respectively. The nirS/amoA, nxrA/amoA, and amoA/(narG + napA) were deemed to be the markers of ammonium oxidation rate (SAOR), specific nitrite oxidation rate (SNOR), and specific nitrate reduction rate (SNRR), respectively. Compared with functional gene quantity data, transcription data (mRNA) introduced into stepwise regression analyses agreed well with nitrogen transformation rates. High nitrogen loading also resulted in the cell viability decreased in R_50_. Nitrogen loadings and operation time both led to a significant variation in cell membrane composition, and unsaturated fatty acids (UFAs) significantly increased in R_30_ (46.49%) and R_50_ (36.34%). High-throughput sequencing revealed that nitrogen loadings increased the abundance of nitrifying bacteria (e.g., *Nitrospira*) and reduced the abundance of denitrifying bacteria (e.g., *Nakamurella*, *Thermomonas*, and *Zoogloea*) through linear discriminant analysis (LDA).

## Introduction

Limited by the volume and high heat capacity of domestic wastewater, many wastewater treatment plants (WWTPs) with special regional climate and seasonal conditions face nitrogen removal problem at low temperatures^1^. Even in some mid-latitude regions such as Roorkee, India and Oregon, USA, wastewater temperatures remain relatively stable at lower than 10 °C in the winter^2,3^. Therefore, to avoid continuous eutrophication in aquatic environments, the development of cold-adapted biological nitrogen removal processes is vital to WWTPs^4^.

The biofilm process is widely recognized to have an excellent nitrification and denitrification capacity (>51% nitrogen removal efficiency) at low temperatures (<10 °C) over four years^5^. An integrated biofilm and activated sludge process is a potential and realizable nitrogen-removal upgrading method for domestic WWTPs in winter^6^. Biofilm packing is capable of immobilizing nitrifier/denitrificans below 8 °C, which has been implemented at various locations^7^. Thus, environmental researchers and engineers have exerted extensive efforts to improve biofilm packing structure and geometry to achieve greater total nitrogen (TN) removal efficiency in recent years^8^. Recently, biochar has been used as a packing material for reactors, based on its large surface area, microporosity, and ability to support microbial biofilm formation^9^. Biochar is a soil amendment to improve N recycling in the soil–plant system. It is a biostable material with pores and crevices on its surface that provide shelter for microorganisms^10^. Biochar addition increases the denitrifying bacteria abundance significantly (nosZ, nirK, and nirS), produced less N_2_O, and promoted nitrate removal in a pilot-scale biochar-packed denitrifying bioreactor^11^. Compared with traditional plastic carrier, biochar packed had the advantage on inorganic nitrogen adsorption, nitrifier/denitrificans immobilization, nitrogen removal rate (NH_4_^+^-N and NO_3_^−^-N), NO_2_ emission, and production price^7,12^.

Nitrogen-loading calculations are important for nitrogen-removal upgrades of WWTPs, and depend on hydraulic residence time or tank volume determination^13^. Nitrogen loading significantly influences NH_4_^+^ and TN removal^14^, which determines wastewater processing capacity. To prevent low nitrogen removal efficiency, it is necessary to set the low nitrogen loading at temperatures lower than 15 °C^15^. Under low nitrogen loading conditions (<15 mg TN/L), functional genes (e.g., amoA, nirS, nxrA, napA, and narG) involved in nitrogen metabolism measured by the quantitative polymerase chain reaction (qPCR) were used to calculate and predict NH_4_^+^, NO_2_^−^, and NO_3_^−^ transformation rates (*p* < 0.05) at 4 °C^16^. However, the rationality of this method for different nitrogen loadings deserves further study, which is one of the main purposes of this research.

In this study, we assessed the effects of nitrogen loading on nitrification and denitrification in biochar packed reactors at 5 °C. The main objectives of our investigation were: (i) to compare the performance of fundamental biochar-packed reactors with different nitrogen loadings; (ii) to assess biofilm performances at different total nitrogen loading, resulting in relative functional gene transcription, in terms of its capability of reducing ammonia nitrogen, nitrate, and nitrite; and (iii) to eventually investigate the association between bioreactor performance, nitrogen transformation rate, and the microbial community.

## Results

### Bioreactor performance

**Figure 1:**
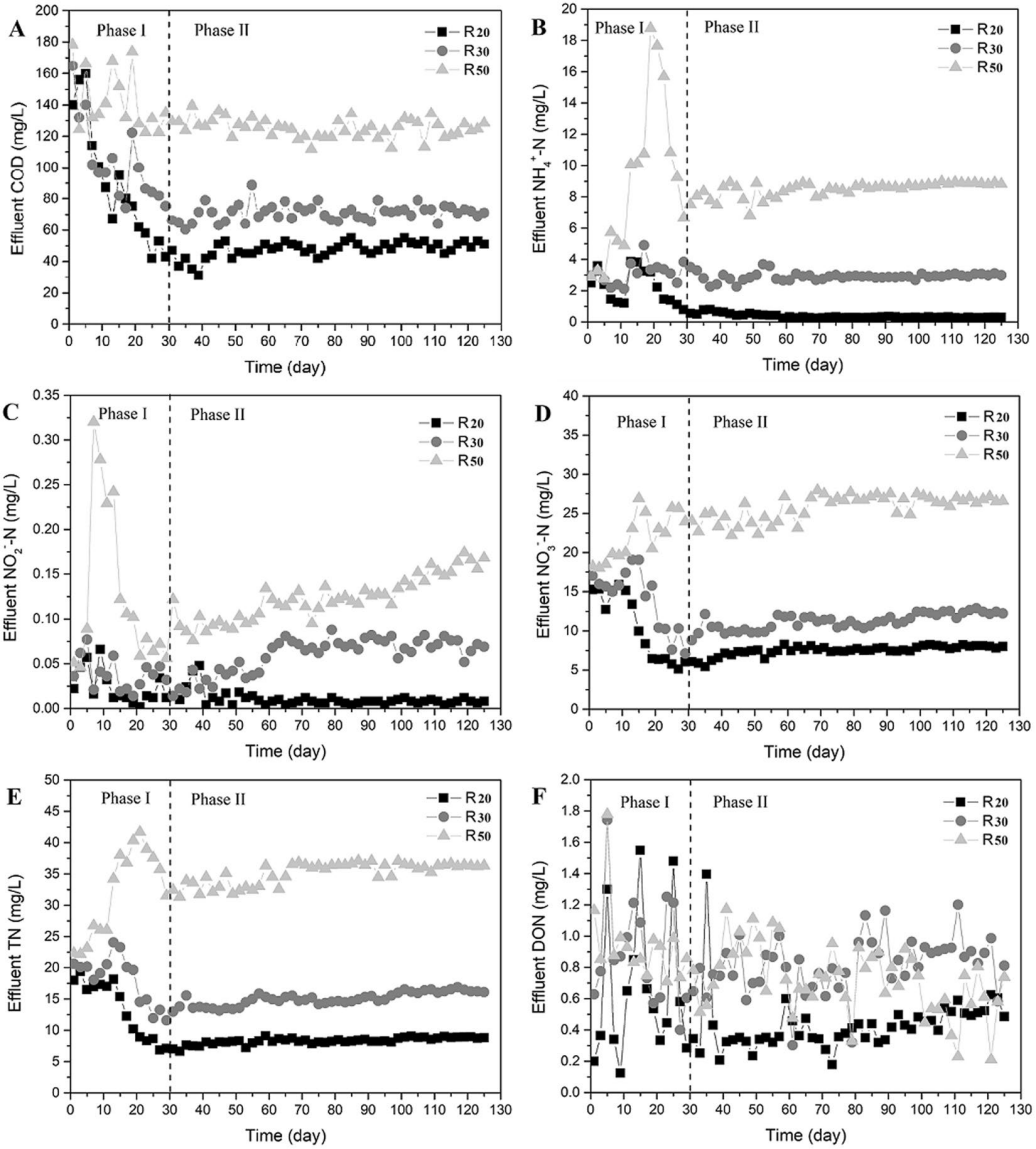
Effluent COD (**A**), NH_4_^+^ (**B**), NO_2_^−^ (**C**), NO_3_^−^ (**D**), TN (**E**) and DON (**F**) in R_20_, R_30_, and R_50_ at 5 °C. Phase I: acclimatization stage; Phase II: operation stability stage.

Figure 1 shows the fundamental effluent quality with different nitrogen loading (20, 30, and 50 mg TN/L) within a 125-day operation period. In phase I, along with a temperature decline (from 20 °C to 5 °C), NO_2_^−^ and NH_4_^+^ showed a short-term accumulation in 50 mg TN/L nitrogen loading. NO_3_^−^ initially accumulated with all nitrogen loadings, and effluent NO_3_^−^ exhibited a greater decrease in R_20_ and R_30_ after the 15^th^ day. In phase II, the biofilm microorganisms adapted to 5 °C, and the fundamental effluent quality remained stable from day 30 to day 125. Effluent TN (NO_3_^−^) concentrations of R_20_, R_30_ and R_50_ declined and stabilized to 8.49 ± 0.55 mg/L (7.81 ± 0.44 mg/L), 15.42 ± 1.13 mg/L (11.74 ± 0.72 mg/L) and 35.69 ± 1.42 mg/L (26.72 ± 0.95 mg/L), respectively, on the 30^th^ day (Fig. 1E). NH_4_^+^ removal efficiency decreased from 89% (R_20_) to 83% (R_50_) as the influent nitrogen loading increased (Fig. 1B). Moreover, effluent DON concentration was found to be lowest in R_20_ (0.44 ± 0.16 mg/L). In this study, the high dissolved oxygen (7.6–8.0 mg/L) severely limited toxic NO_2_^−^ accumulation (<0.2 mg/L) during stabilization (Fig. 1C), even with 50 mg TN/L nitrogen loading. With the increase of influent COD loading (R_20_: 400 mg/L, R_30_: 600 mg/L and R_50_: 1,000 mg/L), effluent COD concentration ranged from 50 mg/L (R_20_) to 125 mg/L (R_50_) (Fig. 1A), and the COD removal rate reached its peak at 88.10% (R_30_).

### Nitrogen transformation rates and quantitative response relationships

**Figure 2:**
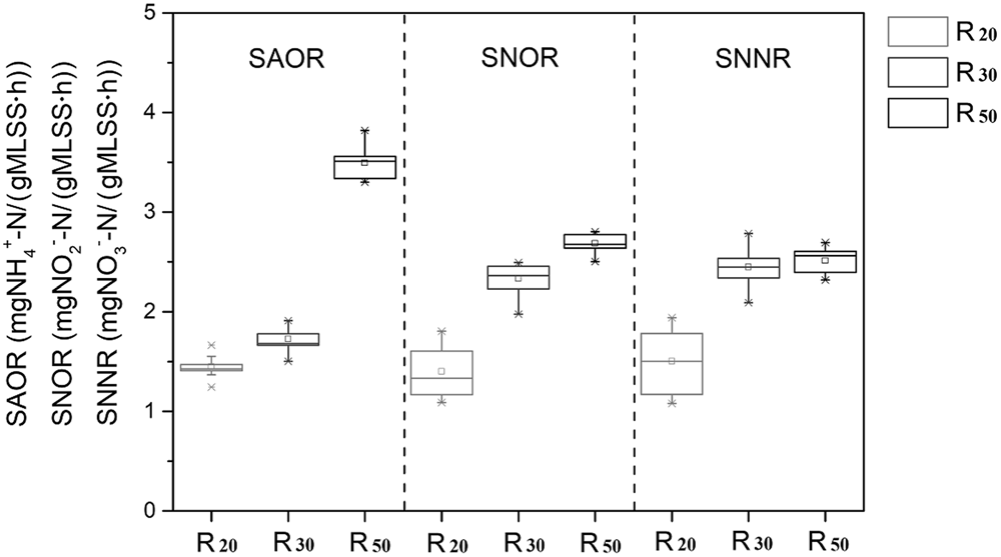
Variation in SAOR, SNOR, and SNRR of R_20_, R_30_, and R_50_ during Phase II (>day 30) with error bars (stdev.), *n* = 17. Detailed values with sampling times are available in the Supplementary Information.

The main forms of nitrogen metabolism at 5 °C, nitrification and denitrification rates were monitored with the measurement of SAOR, SNOR and SNRR (Fig. 2, see Supplementary Fig. S1). During the stable operation (phase II, >30^th^ day, n = 17), SAOR was represented as 141% and 103% higher in R_50_ (3.49 ± 0.33 mgNH_4_^+^/(gMLSS·h)) than that in R_20_ (1.45 ± 0.21 mgNH_4_^+^/(gMLSS·h)) and R_30_ (1.72 ± 0.19 mgNH_4_^+^/(gMLSS·h)). SNNR reached a maximum value of 2.51 ± 0.2 mgNO_3_^−^/(gMLSS·h) in R_50,_ which was a little more than in R_30_ (2.44 ± 0.27 mgNO_3_^−^/(gMLSS·h)). SNOR showed the same trend as SNNR, and no nitrite accumulation (<0.35 mg/L) phenomenon occurred in the effluent of R_20_, R_30_, or R_50_ (Fig. 1C).

As shown in Table 1, functional gene quantity/transcription and nitrogen transformation rates (SAOR, SNOR, and SNNR) were used as variables, which were introduced into stepwise regression analyses. Under the condition of 20 mg TN/L nitrogen loading, quantitative response relationships showed that nirS/amoA, nxrA/amoA, and amoA/(narG + napA) were the respective key functional gene groups determining NH_4_^+^ oxidation (mgNH_4_^+^/(gMLSS·h)), NO_2_^−^ oxidation (mgNO_2_^−^/(gMLSS·h)), and NO_3_^−^ (mgNO_3_^−^/(gMLSS·h)) reduction at 5 °C. However, with the increase of nitrogen loading, nitrogen transformation rates became unsatisfactory and unreasonable in R_30_ and R_50_ (*p* > 0.05) according to functional gene abundance (Table 1). Compared with functional gene quantity data, functional genes transcription data (mRNA) introduced into stepwise regression analyses agreed well with nitrogen transformation rates in terms of SAOR, SNOR and SNNR (*p* < 0.05).

**Table 1:** Quantitative response relationships between nitrogen transformation rates and functional gene abundance/expression in R_20_, R_30_, and R_50_.

	Stepwise regression eqs.(DNA, 7–125 day, n = 5)	R^2^	P	Stepwise regression eqs (RNA, 7–125 day, n = 5)	R^2^	P
R_20_	SAOR = 0.2599 (nirS/amoA) + 1.572	0.962	0.030	SAOR = −2.401 (nirS/amoA) + 2.620	0.955	0.004
	SNOR = −1.219 (nxrA/amoA) + 5.484	0.996	0.002	SNOR = −1.277 (nxrA/amoA) + 2.917	0.912	0.045
	SNNR = 2.678 (amoA/(narG + napA)) + 1.131	0.979	0.010	SNNR = 0.104 (amoA/(narG + napA)) + 1.374	0.941	0.030
R_30_	SAOR = 0.007 (nirS/amoA) + 1.477	0.173	0.486	SAOR = −1.431 (nirS/amoA) + 2.203	0.989	0.005
	SNOR = −76.335 (nxrA/amoA) + 3.617	0.497	0.184	SNOR = −7.174 (nxrA/amoA)) + 4.422	0.972	0.020
	SNNR = −4.425 (amoA/(narG + napA)) + 3.807	0.821	0.094	SNNR = −0.159 (amoA/(narG + napA)) + 2.681	0.992	0.004
R_50_	SAOR = 0.0068 (nirS/amoA) + 3.457	0.085	0.632	SAOR = −0.632 (nirS/amoA) + 3.696	0.971	0.020
	SNOR = −5.113 (nxrA/amoA) + 2.803	0.613	0.117	SNOR = 1.845 (nxrA/amoA) + 3.135	0.976	0.020
	SNNR = −0.965 (amoA/(narG + napA)) + 2.723	0.386	0.263	SNNR = −0.185 (amoA/(narG + napA)) + 1.922	0.964	0.030

Significant correlation: p < 0.05; non-significant correlation: p > 0.05.

### Integrity and composition of cell membrane

The cell membrane integrity rate indicated by the cell viability and fluorescence microscopy photographs were determined through BacLight Staining^7^ (Table 2 and Fig. S5). Specifically, the cell viability of R_20_ showed the maximum value (R_20_, day 15: 76.98%; R_20_, day 35: 93.54%; R_20_, day 75: 91.85%; R_20_, day 125: 93.62%). By comparison, the cell viability of R_50_ showed the minimum value (R_20_, day 15: 47.85%; R_20_, day 35: 46.48%; R_20_, day 75: 50.83%; R_20_, day 125: 42.02%). In phase II (>day 35), the cell membrane integrity rate tended to stabilize (R_20_: > 90%, R_30_: 55–85%, R_50_: 40–50%).

**Table 2:** Cell membrane viability changes with different nitrogen loadings, reactors, and sampling times.

Time	R_20_	R_30_	R_50_
15^th^ day	76.98%	60.35%	47.85%
35^th^ day	93.54%	56.71%	46.48%
75^th^ day	91.85%	82.79%	50.83%
125^th^ day	93.62%	70.20%	42.02%

^*^Measured values for cell viability were the average value ± standard deviation, n = 20.

**Figure 3:**
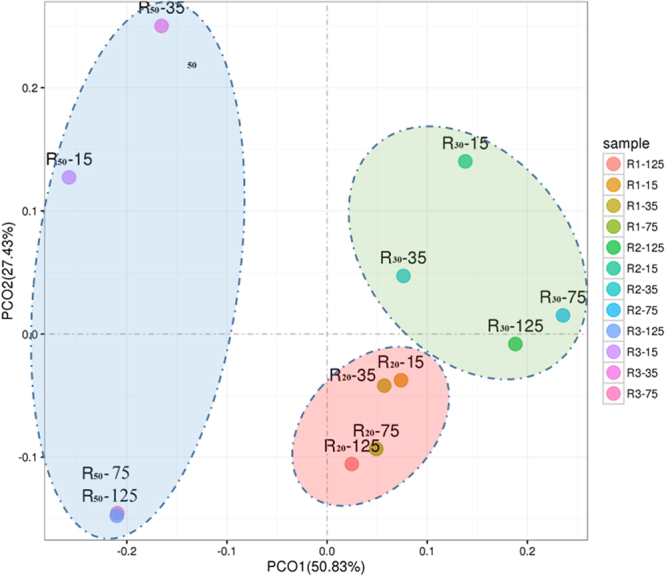
Principal co-ordinate analysis of PLFAs in R_20_, R_30_, and R_50_ with different sampling times. The number after the dash represents sampling day.

The phospholipid fatty acid (PLFA) constructions of R_20_, R_30_, and R_50_ were measured on days 15, 35, 75, and 125 (see Supplementary Table S4). Principal coordinate analysis (PCoA) was performed for the unweighted and weighted UniFrac distances to determine if PLFA construction shifted with different sampling times and nitrogen loadings. Significant variations existed in PLFA construction between three different nitrogen loadings (Fig. 3). This result confirms that nitrogen loading is an important factor in PLFA construction. Moreover, the PLFA construction diversity significantly changed with respect to operation time, particularly in 50 mg TN/L nitrogen loading. Unsaturated fatty acids (UFAs) (e.g., 18:2 cis 9,12, 20:4 cis 5,8,11,14) significantly increased in R_30_ (46.49%) and R_50_ (36.34%) compared with R_20_ (28.57%) on day 125 (Table S4).

### Microbial community

The microbial community was investigated with a 16 S rRNA gene-based Illumina MiSeq sequencer to fully understand the impacts of long-term biochar packed reactors operation at 5 °C (see Supplementary Tables S2 and S3). All samples of high-throughput Illumina sequencing were normalized to 9,648 OTUs, corresponding to the sample with the least number of reads. At the genus level, the OTUs, which showed up to 2% relative abundance, were selected to generate the heat map (Fig. 5A). After day 30 (phase II), when the reactors were at the peak of their nitrogen removal ability, the bacterial communities of the same reactor were similar on days 35, 75, and 125. Proteobacteria was the most abundant phylum in R_20_, R_30_, and R_50_, accounting for 25.12% in R_20_, 24.70% in R_30_, and 39.98% in R_50_ on day 125. The other dominant phyla in R_20_, R_30_, and R_50_ were Actinobacteria (R_20_: 11.04%; R_30_: 23.35%; R_50_: 4.80%) and Bacteroidetes (R_20_: 11.4%; R_30_: 9.86%; R_50_: 8.68%) on day 125. At the genus level, *Pseudomonas* (R_20_: 2.06%; R_30_: 1.66%; R_50_: 1.08%), *Janthinobacterium* (R_20_: 2.98%; R_30_: 4.51%; R_50_: 0.69%), *Arthrobacter* (R_20_: 2.32%; R_30_: 12.66%; R_50_: 1.76%), and *Flavobacterium* (R_20_: 3.68%; R_30_: 4.20%; R_50_: 2.62%) were most abundant in all reactors on the 125^th^ day. It is important to note that *Serratia* (R_20_: none detected; R_30_: 1.84%; R_50_: 8.42%) only achieved high abundance when nitrogen loading >20 mg TN/L on day 125.

**Figure 4:**
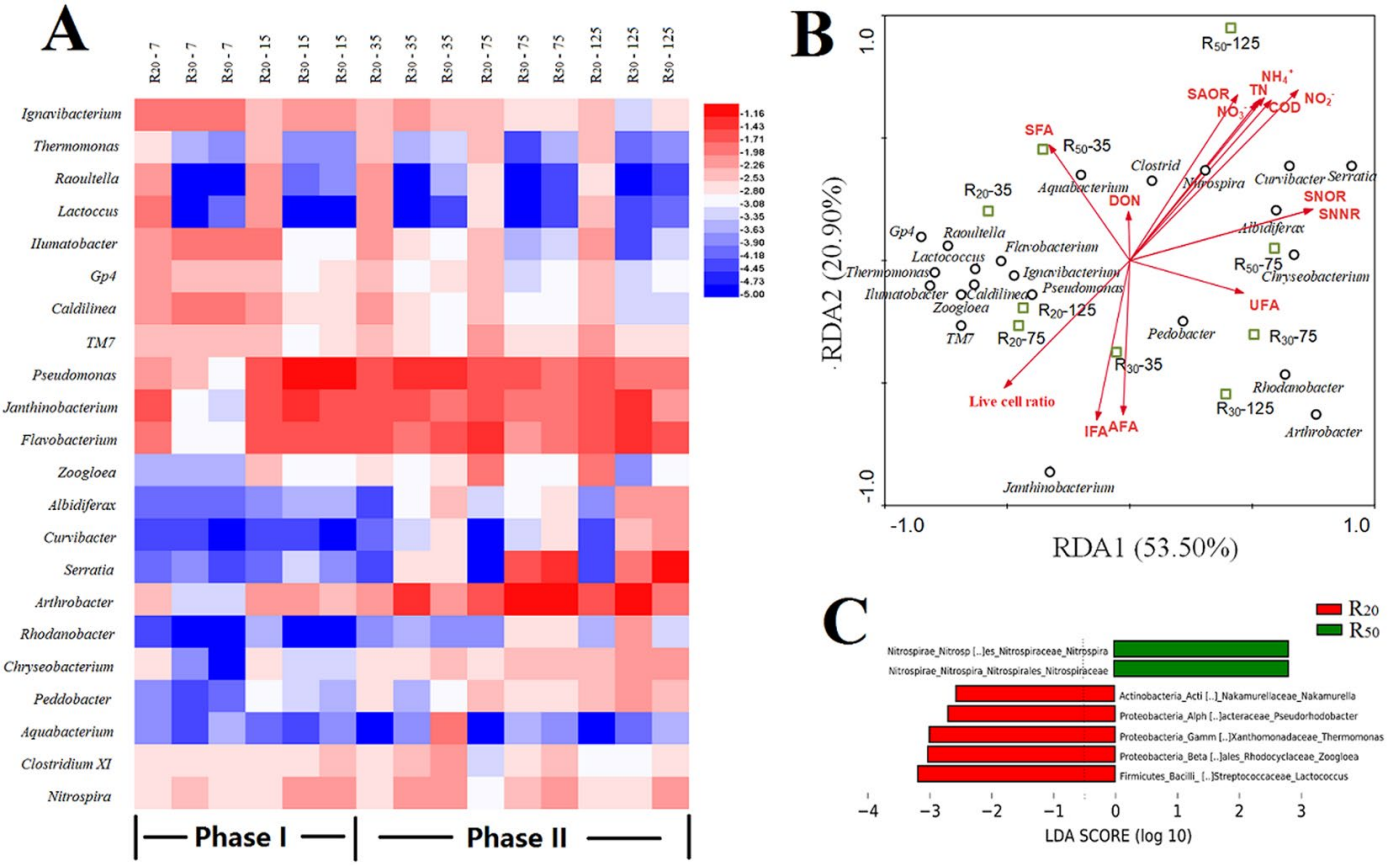
Bacterial community structures (**A**), redundancy analysis (**B**), and linear discriminant analysis (**C**) during different periods of R_20_, R_30_, and R_50_ at 5 °C at the genus level. The color bar indicates the range of the percentage of a genus in a sample, based on the color key (log10 scale) in the right corner.

**Figure 5:**
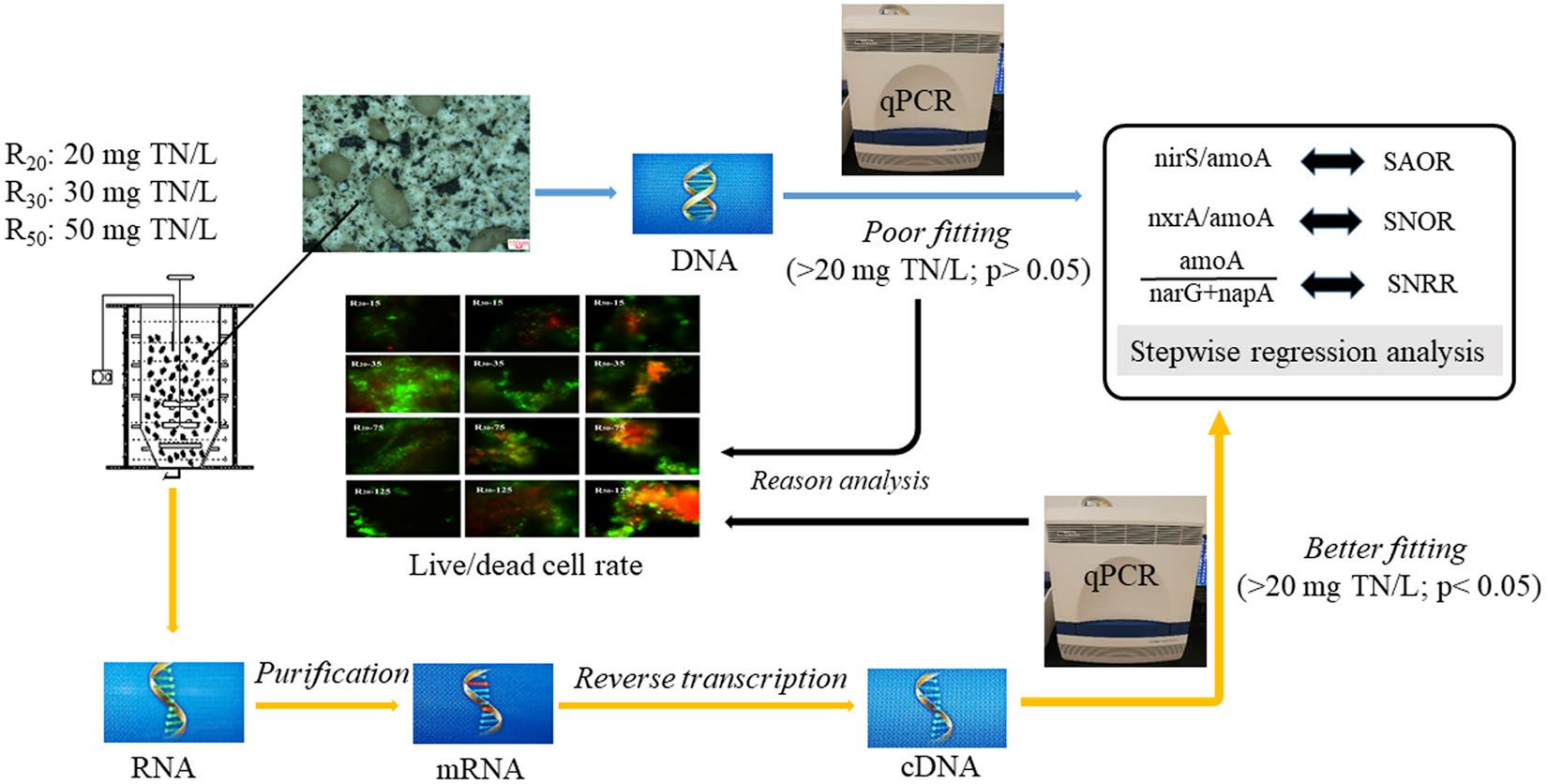
Inter-group significant differences and reason analysis of nitrogen transformation rates (SAOR, SNOR and SNNR) and functional gene abundance/expression. qPCR = quantitative polymerase chain reaction; SAOR = specific ammonium oxidation rate; SNOR = specific nitrite oxidation rate; SNRR = specific nitrate reduction rate; TN = total nitrogen. Significant correlation: p < 0.05; non-significant correlation: p > 0.05.

Redundancy analysis (RDA) was used to evaluate the relationship between reactor environmental factors and bacterial population structure (>2‰) on days 35, 75, and 125 by relative abundance at the genus level (Fig. 5B). RDA1 and RDA2 explained 53.50% and 20.90% of the total variance, respectively. The distribution of bacterial community structures was clearly divided into three groups, R_20_, R_30_, and R_50_, because the angles between adjacent groups were nearly perpendicular, indicating that they were unrelated (Fig. 5B). Notably, *Nitrospira* showed a positive correlation with NH_4_^+^ (Pearson test, *p* = 0.036), NO_3_^−^ (Pearson test, *p* = 0.036), TN (Pearson test, *p* = 0.037), SAOR (Pearson test, *p* = 0.047), SNOR (Pearson test, *p* = 0.026), and SNNR (Pearson test, p = 0.025). *Curvibacter* had a positive correlation with NO_2_^−^ (Pearson test, *p* = 0.007), SNOR (Pearson test, *p* = 0.046), and SNNR (Pearson test, *p* < 0.048). UFAs showed positive correlation with SNOR (Pearson test, *p* = 0.046) and SNNR (Pearson test, *p* = 0.046). Linear discriminant analysis (LDA) determines the features most likely to contrast change between classes by coupling standard tests for statistical significance with additional tests encoding biological consistency and effect relevance^17^. As shown in Fig. 4C, nitrogen loading increased the abundance of nitrifying bacteria (e.g., *Nitrospira*) and reduced the abundance of denitrifying bacteria (e.g., *Nakamurella*, *Thermomonas*, and *Zoogloea*).

## Discussion

Low temperatures (5 °C) had little effect on ammonia nitrogen transformation of biofilm, and the high NH_4_^+^ removal rate of biofilm (>98%) was usually reported at 4–12 °C^16,18,19^. The considerable effluent COD (>50 mg/L) of R_30_ and R_50_ implied an inadequate biofilm aerobic oxidation and denitrification ability at a low temperature^20^. Inadequate biofilm denitrification ability contributed to NO_3_^−^ accumulation and the unsatisfactory TN removal rate of R_30_ and R_50_, which was also reported in an activated sludge system (25% TN removal rate at 10–15 °C) at low temperatures^21,22^. Under the same temperature and nitrogen loading, R_20_ represents a higher NH_4_^+^ (98%) and TN (57%) removal rate than conventional activated sludge (NH_4_^+^ removal rate: 92%; TN removal rate: 33%)^23^. Biochar addition promoted effluent NH_4_^+^ and TN removal, which resulted from nitrifier/denitrificans immobilization below 8 °C and the increase of functional genes abundance (amoA, napA, nxrA, nosZ, narG, nirK, and nirS)^7,24^. Moreover, this might relate to many different functional groups on the surface of biochar, such as nitro, chloro, hydroxyl, amine, carbonyl and carboxylic^25^. Although more than 20 mg TN/L nitrogen loading facilitated a biofilm denitrification problem at 5 °C, a biochar packed reactor was helpful for DON removal with 20 mg TN/L nitrogen loading compared with traditional activated sludge reactors^23^. DON removal improvement from traditional activated sludge (3.45 ± 0.41 mg/L) to biochar (0.44 ± 0.16 mg/L) is essential in WWTPs because nitrogenous compounds are a potential threat to aquatic ecosystems^23^.

According to previous studies^26–32^, functional gene abundance can be calculated and effectively introduced into stepwise regression analyses with nitrification and denitrification transformations (e.g., SAOR, SNOR and SNNR). Specifically, for two consecutive steps of nitrification and denitrification, the amoA and nirS genes are thought to be NH_4_^+^ to NO_2_^−^ oxidation and NO_2_^−^ to NO reduction markers, respectively^26,27^. Low NO_2_^−^ concentration is beneficial to NH_4_^+^ transformation, because NO_2_^−^ has toxic effects on ammonia-oxidizing bacteria^28,29^. Similarly, nxrA is regarded as the NO_2_^−^ to the NO_3_^−^ oxidation marker involved in the nitrite oxidation process^30^. Both narG and napA are regarded as marker genes for NO_3_^−^ into NO_2_^−^ reduction, and are responsible for the first denitrification step. Generally, narG is dominant under anoxic and napA under oxic conditions^31,32^. Therefore, nirS/amoA, nxrA/amoA, and amoA/(narG + napA) were deemed to be the markers of SAOR, SNOR, and SNNR at 4 °C^16^.

According to the functional gene quantity and transcription data (see Supplementary Figs S2 andS3), we performed stepwise regression analyses between qPCR data and nitrogen transformation rates (SAOR, SNOR, and SNNR) on days 7, 15, 35, 75, and 125. As shown in Fig. 5 and Table 1, when functional gene quantity and transcription data (mRNA) were both introduced into stepwise regression analyses, the results agreed well with nitrogen transformation rates in 20 mg TN/L nitrogen loading (Pearson test, *p* < 0.05), whereas when only functional gene transcription data were introduced into stepwise regression analyses, the results did not agree well with nitrogen transformation rates in R_30_ and R_50_ (Pearson test, *p* > 0.05). The specific reasons are as follows. Extractive DNA originated from both live and dead cells, but extractive RNA only came from live cells. We hypothesize that DNA from dead cells contributed to the inaccuracy in the fitting between functional gene abundance and nitrogen transformation rates. We measured the cell viability in different nitrogen loadings with different sampling times, and the results showed obvious variations in the cell viability between different nitrogen loadings. Limited by cell viability variation, functional gene transcription data (mRNA) better fitted nitrogen transformation rates than functional gene quantity data, especially in R_30_ and R_50_.

Low temperatures contribute to cell membrane transfer rate decreases^33^, and substrate mass transfer rate was another important factor for nitrogen transformation. For example, substrate (e.g., NH_4_^+^) mass transfer depends on an ammonia transporter protein on PLFA. Meanwhile, nitrification and denitrification enzymes and electron transport of coenzymes (MQH_2_ and QH_2_) are located in the cell membrane^34^. The results showed that nitrogen loading and operation time both changed PLFA construction. From the observations shown in Fig. 4B, UFAs were significantly higher in R_30_ (46.49%) and R_50_ (36.34%) than in R_20_ (28.57%), which enhanced the mass transfer efficiency of cells, and is necessary for efficient mass transfer at low temperature^35^. Moreover, Eliška, R. & Kateřina, H found that nitrogen loadings result in a PLFA content change^36^, which is often used to indicate the microbial community^23,33,35^. The cell viability decreased as the nitrogen loading increased, which might result from effluent metabolites, extracellular polymeric substances, and the microbial community structure. However, microbial community structure also plays an important role in nitrification and denitrification at low temperatures^37^.

Biochar promoted the adsorption of NH_4_^+^ and NO_3_^−^^12,38^, increased the abundance of nitrifying bacteria and the NO_3_^−^ removal rate^39,40^, and changed microbial community construction for better nitrogen removal^41,42^. *Pseudomonas*, *Janthinobacterium*, *Arthrobacter*, and *Flavobacterium* occupied the highest abundance in R_20_, R_30_, and R_50_, which agreed with the activated sludge system at low temperatures^23,43^. Among them, *Pseudomonas*, *Arthrobacter*, and *Flavobacterium* were enriched in biochar with a high ammonification ability in the soil^44,45^. This bacterial genus enrichment might be a result of specific biochar surface characteristics, which concurrently partition microorganism spatial distribution, and prompt microorganism absolute abundance, as well as microbial community’s structural stability^46^. *Serratia* only occupied high abundance in R_30_ and R_50_, which could produce extracellular polymeric substances^47^ (e.g. protein, polysaccharide, nucleic acid). Protein and nucleic acid are an important DON source after cell death from hydrolysis^22^. *Curvibacter* had positive correlation with NO_2_^−^, SNOR, and SNNR in Fig. 5B, which could use both nitrate and nitrite as an electron acceptor under both aerobic and anoxic conditions^48,49^. DON had no significant correlation with any bacterial genus, which was consistent with our previous study^22^, and no single bacterial genus could degrade DON effectively.

In conclusion, we studied the impacts of nitrogen loading on nitrification and denitrification in biochar packed reactors at 5 °C. Three significant findings show the novelty of this research. (1) Biochar represents a high NH_4_^+^, (TN, and DON) removal rate at 5 °C. High nitrogen loading resulted in an NH_4_^+^ (TN) removal efficiency decline from 98% (57%) to 83% (29%), but biochar represented a higher NH_4_^+^, TN and DON removal rate than conventional activated sludge. (2) Nitrogen loading has effect on cell viability, UFAs, and microbial community at 5 °C. The cell viability, UFAs and microbial community under different nitrogen loading is relatively less researched compared to other nitrogen-removal research at 5 °C. (3) Compared with functional gene quantity data, the transcription data (mRNA) introduced into stepwise regression analyses agreed agrees well with nitrogen transformation rates. This is an area that needs more research, but based on this research and the research that has preceded it^16,46^, it is well worth pursuing.

## Materials and Methods

### Bioreactor

Three SBRs (R_20_, R_30_, and R_50_) fed with different total nitrogen loading (20, 30, and 50 mg/L) were operated at 5 ± 0.1 °C (incubator: Sanyo Electric) with a working volume of 2 L. Seeding sludge was obtained from the aeration tank (suspended solids concentration about 4,400 mg/L) of a municipal wastewater treatment plant in Nanjing, China. Biochar was made from local rice husk charred at temperatures 600–800 °C for 8 h, and had a diameter of 0.5–3.0 mm and a specific surface area of 155.51 m^2^/g. This biochar was added to the experimental reactor (10 g/L). Effluent was discharged from the middle port of the reactor with a volumetric exchange ratio of 50%. The hydraulic retention time of the SBRs was 12 h and the feeding time, reaction time, settling time, and anoxic time were 0.5 h, 10 h, 0.5 h, and 1 h, respectively. The reactors were generally operated with fixed mixed liquor suspended solids (MLSS) with a concentration of 4,000 mg/L and an oxygen concentration of 7.6–8.0 mg/L.

### Synthetic wastewater

The synthetic wastewater took glucose, NH_4_Cl, and KH_2_PO_4_ as carbon, nitrogen, and phosphorus sources, respectively, and the mass ratio of C:N:P was 100:5:1 (Table 3). Furthermore, the synthetic wastewater was set with different TN loadings (20, 30 and 50 mg/L). One liter of synthetic wastewater contained 0.6 mL (R_20_), 0.9 mL (R_30_), and 1.5 mL (R_50_) of metals solution, and one liter of the metals solution contained 11 g CaCl_2_·2H_2_O, 1.5 g FeCl_3_·6H_2_O, 0.15 g H_3_BO_3_, 0.03 g CuSO_4_·5H_2_O, 0.18 g KI, 0.12 g MnCl_2_·4H_2_O, 0.06 g Na_2_MoO_4_·2H_2_O, 0.12 g ZnSO_4_·7H_2_O, 0.15 g CoCl_2_·6H_2_O, and 10 g EDTA. Furthermore, NaHCO_3_ was added to keep the alkalinity in the SBRs in the range of pH = 7.0 ± 0.5.

**Table 3:** Experimental conditions of R_20_, R_30_ and R_50_.

Experimental phase (d)		Temperature (°C)	Feeding condition (C:N:P = 100:5:1)			
				R_20_	R_30_	R_50_
Phase I	1–5	20	COD (mg/L)	400	600	1,000
	6–10	15	TN (mg/L)	20	30	50
	11–15	10	TP (mg/L)	4	6	10
	16–30	5	Metals solution (mL/L)	0.6	0.9	1.5
Phase II	30–125	5	pH	7.0 ± 0.5		

*One liter of the metals solution contained: 11 g CaCl_2_·2H_2_O, 1.5 g FeCl_3_·6H_2_O, 0.15 g H_3_BO_3_, 0.03 g CuSO_4_·5H_2_O, 0.18 g KI, 0.12 g MnCl_2_·4H_2_O, 0.06 g Na_2_MoO_4_·2H_2_O, 0.12 g ZnSO_4_·7H_2_O, 0.15 g CoCl_2_·6H_2_O, and 10 g EDTA. NaHCO_3_ was added to keep the alkalinity in the range of pH = 7.0 ± 0.5.

### Fluorescent staining

The AS samples were obtained from running SBRs (reaction time). Immediately, the samples were stained using a 2 μL LIVE/DEAD BacLight bacterial viability kit, and were then observed with an epifluorescence microscope after the samples had been diluted 10 times. The BacLight bacterial viability kit consists of two stains, propidium iodide (PI) and SYTO9, which were both used to stain the nucleic acids. Green fluorescing SYTO9 was used to permeate all cells, which enabled us to acquire total cells counts, whereas red fluorescing PI only enters cells with damaged cytoplasmic membranes. The obtained microscopic images were visualized with Image J software (a Java-based image processing program, National Institutes of Health, USA). After taking an average of 20 samples, we counted the live/dead cells rate^7^.

### Microorganism molecular analysis

#### DNA and RNA extraction

DNA extraction of 0.5 g from each sample was performed using a FastDNA® SPIN Kit for soil (MP Biomedicals, OH, USA) following the manufacturer’s protocol. An E.Z.N.A. TM Cycle-Pure Kit (Omega Bio-tek Inc., USA) was used to extract and purify total genomic DNA. RNA extraction (0.5 g suspended solids per sample) was performed using a *TransZol* Up Plus RNA Kit (TransGen Biotech, China). RNA samples were purified with *MagicPure*^TM^ RNA Beads (TransGen Biotech, China), and reverse transcription to cDNA through an EasyScript^®^ One-Step gDNA Removal and cDNA Synthesis SuperMix (TransGen Biotech, China). The amount and purity of DNA, RNA, and cDNA were determined using a NanoDrop® Spectrophotometer ND-1000 (Thermo Fisher Scientific, MA, USA) based on an absorbency of A260 and a ratio of A260:A280. Extracted DNA, RNA, and cDNA were checked by 2% agarose gel electrophoresis and stored at −80 °C.

#### Quantitative PCR

The abundance of bacterial 16 S rRNA, and nitrogen functional genes, including ammonia monooxygenase (amoA), periplasmic nitrate reductase (napA), nitrite oxidoreductase (nxrA), nitrite reductase (nirS), nitrite reductase (nirK), and membrane-bound nitrate reductase (narG) were identified (see Supplementary Table S1). Further, the related transcription stations of these genes were measured with quantified cDNA. The qPCR used a 7500 Real Time PCR System (Applied Biosystems) with the fluorescent dye SYBR-Green approach, which was employed in rRNA and functional gene amplification. Related statistical analyses were completed according to Pang *et al*.^14^ and Srinivasan *et al*.^50^.

#### High-throughput sequencing of 16 S rRNA gene

PCR amplification was carried out using the forward primer (5′-ACT CCT ACG GRA GGC AGC AG-3′) and reverse primer (5′-TCT CAN VGG GTA TCT AAT CC-3′) for the V3-V4 hypervariable region of the 16 S rRNA gene^51^. PCR was performed in a Veriti^®^ 96-Well Thermal Cycler (Applied Biosystems, USA). The quadruplicate PCR reactions for each sample preparation were purified again using the E.Z.N.A.^TM^ Cycle-Pure Kit (Omega Bio-tek Inc., USA) and stored at −80 °C. About 500 ng of the purified PCR product for each sample was mixed and sequenced on an Illumina MiSeq sequencer. After sequencing, Python scripts were written to perform quality filtering of the raw reads with the Sickle and Mothur programs to remove low-quality sequences and reduce noise. The filtered sequences were assigned to taxa by the RDP classifier. The detailed data analysis is available in the Supplementary Methods and Results.

### Other methods

Effluent samples were filtered through 0.45 µm cellulose acetate membranes (Anpel Co., Ltd., China), stored in the dark, kept at −20 °C prior to the experiments, and analyzed within one week of collection. The analyses of COD, NH_4_^+^, NO_3_^−^, NO_2_^−^, and TN were determined as per the Standard Methods^52^. The differences between TN and total inorganic nitrogen (NH_4_^+^, NO_2_^−^, and NO_3_^−^) was determined to be DON^53^. The determination of SAOR, SNOR, and SNRR measurements followed Wang *et al*.^54^. The PLFA extraction and analysis method was according to our previous study (2018).

## Electronic supplementary material

Supplementary Material
