# Supplementary material for: Nitrogen loading effects on nitrification and denitrification with functional gene quantity/transcription analysis in biochar packed reactors at 5 °C
Source: Sci Rep. 2018 Jun 29;8:9844. doi: 10.1038/s41598-018-28305-0 (PMC6026168; doi:10.1038/s41598-018-28305-0)
Supplement: Supplementary file 1 — Supplementary Material [file 41598_2018_28305_MOESM1_ESM.doc]

**Supplementary Material for:**

**Nitrogen loading effects on nitrification and denitrification with functional gene quantity/transcription analysis in biochar packed reactors at 5 °C**

Su He, Lili Ding, Yao Pan, Haidong Hu, Lin Ye, Hongqiang Ren*

**State Key Laboratory of Pollution Control and Resource Reuse, School of the Environment, Nanjing University, Nanjing 210023, Jiangsu, PR China**

***Corresponding authors: Hongqiang Ren, Tel: +86 25 89680512, Fax: +86 25 89680569, E-mail address:** [**hqren@nju.edu.cn**](mailto:hqren@nju.edu.cn)

This document consists of 13 pages, 2 materials and methods, 4 tables, 5 figures and references.

**Table S1:** Primers, protocols and parameters of target genes in qPCR analysis.

**Table S2**: Bacterial taxonomic assignment at the phylum level with Illumina MiSeq sequencing.

**Table S3**: Bacterial taxonomic assignment at the genus level with Illumina MiSeq sequencing.

**Table S4**. The whole PLFA profiles (%) of the microorganism in R_20_, R_30_ and R_50_ at 5 °C.

**Fig. S1** Variations of SAOR, SNOR, and SNRR of R_20_, R_30_ and R_50_ at 5 °C.

**Fig. S2** Relative functional genes abundance of amoA, napA, nirK, nirS, nirG and nxrA during different periods of R20, R30 and R50 at 5 °C

**Fig. S3** Fold changes in amoA, napA, nirK, nirS, nirG and nxrA expression as compared to R4-7^th^ day sample during different periods of R_20_, R_30_ and R_50_ at 5 °C.

**Fig. S4** PCR amplification functional gene in the seeding sludge. Marker, DNA Marker DL 2000.

**Fig. S5** Fluorescence microscopic images in R_20_, R_30_ and R_50_ with different sampling times. The samples were viewed under × 100 magnification. Live cells are stained green, dead cells are stained red. The number after dash represents sampling day.

**Materials and methods**

**Illumina MiSeq Data Analysis**

Sequencing data were estimated using Sickle to remove the bases of low quality (Q<25) and any sequences with more than one N (<https://github.com/najoshi/sickle>). The Mothur program was used for sequence demultiplexing and filtration. General steps are as follows: 1) Sequences were demultiplexed based on barcodes. 2) Read1 and Read2 were combined into contigs (parameter: minoverlap=10, maxhomop=8, maxambig=0). 3) Sequences which were not aligned to the V3V4 region were discarded. 4) Sequences with no more than three different bases were pre-clustered into one sequence. 5) Chimera was detected by comparing sequences with reference databases using the chimera.uchime command packaged in Mothur. 6) The 16S rRNA gene sequences were classified into operational taxonomic units (OTUs) at 3% cutoff (or 97% similarity). The filtered sequences were classified by the stand-alone RDP classifier.

**References**

Zhu, Y. *et al*. Physicochemical characteristics and microbial community evolution of biofilms during the start-up period in a moving bed biofilm reactor. *Bioresour Technol*. **180**, 345–351 (2015).

Coatsa, E.R., Brinkman, C.K. & Lee, S. Characterizing and contrasting the microbial ecology of laboratory and full-scale EBPR systems cultured on synthetic and real wastewaters. *Water Res*. **108**, 124–136 (2017).

**Quantitative PCR**

An abundance of bacterial 16S rRNA; nitrogen functional genes, including ammonia monooxygenase (amoA), periplasmic nitrate reductase (napA), nitrite oxidoreductase (nxrA), nitrite reductase (nirS), nitrite reductase (nirK) and membrane-bound nitrate reductase (narG) were identified. Quantitative polymerase chain reaction (qPCR) using a 7500 Real Time PCR System (Applied Biosystems) with the fluorescent dye SYBR-Green approach was employed in functional gene amplification. Method and related statistical analyses was according to Pang et al (2015).

**References**

Pang, Y., Zhang, Y., Yan, X. & Ji, G. Cold temperature effects on long-term nitrogen transformation pathway in a tidal flow constructed wetland. *Environ Sci Technol*. **49**, 13550-13557 (2015).

**Table S1:** Primers, protocols and parameters of target genes in qPCR analysis

| **Target prokaryote** | **Target gene** | **Sequence (5’-3’) of parameter pairs** | **bp** | **R^2^** | **Ef%** | **Thermal program** | **References** |
| --- | --- | --- | --- | --- | --- | --- | --- |
| Total bacteria | 16S rRNA | 341F: CCTACGGGAGGCAGCAG  534R: ATTACCGCGGCTGCTGG | 194 | 0.998 | 110.945% | 5 min at 95 °C, 40 cycles of 30 s at 95 °C, 30 s at 60 °C, and 40 s at 72 °C | He et al., 2007 |
| AOB | *amoA* | amo598f: GAATATGTTCGCCTGATTG  amo718r: CAAAGTACCACCATACGCAG | 120 | 0.972 | 100.055% | 5 min at 94 °C, followed by 40 cycles of 15 s at 94 °C,30s at 60°C, and 15 s at 72 °C | Dionisi et al.,2002 |
| Denitrifying bacteria | *nirS* | nirScd3aF:GT(C/G)AACGT(C/G)AAGGA(A/G)AC(C/G)GG  nirSR3cd：GA(C/G)TTCGG(A/G)TG(C/G)GT  CTTGA | 425 | 0.973 | 89.749% | Pre-heating at 50 °C for 2 min, pre-denaturation at 95 °C for 10 min, 95 °C for 15 s, 57 °C for 30 s, extension 72 °C for 30 s | Throback et al., 2004 |
| Denitrifying bacteria | *nirK* | nirK583F ：TCATGGTGCTGCCGCGKGACGG  nirK909R：GAACTTGCCGGTKGCCCAGAC | 326 | 0.985 | 101.530% | 95 °C for 10 min, denaturation at 95 °C for 15 s, annealing at 64 °C for 40 s, and extension at 72 °C for 30 s | Yan et al., 2003 |
| Dissimilarity nitrite reducing bacteria | *narG* | narG-f: TCGCCSATYCCGGCSATGTC  narG-r：GAGTTGTACCAGTCRGCSGAYTCSG | 173 | 0.991 | 87.532% | 10min at 95 °C, followed by 40 cycles of 30s at 95 °C, 30s at 58 °C, and 30s at 72 °C | Bru et al., 2007 |
| NOB | *nxrA* | F1nxrA: CAGACCGACGTGTGCGAAAG  R1nxrA: TCYACAAGGAACGGAAGGTC | 322 | 0.992 | 96.697% | Pre-heating at 50 °C for 2 min, pre-denaturation at 95 °C for 10 min, 95 °C for 15 s, 58 °C for 30 s, 72 °C for 30 s | Zhi et al., 2014 |
| Dissimilarity nitrite reducing bacteria | *napA* | napAV17F: TGGACVATGGGYTTYAAYC  napA 4R: ACYTCRCGHGCVGTRCCRCA | 152 | 0.964 | 97.669% | 10min at 95 °C, followed by 40 cycles of 30s at 95 °C, 30s at 58 °C, and 30s at 72 °C | Bru et al., 2007 |

**References**

He, S., Gall, D.L. & McMahon, K.D. "Candidatus accumulibacter" population structure in enhanced biological phosphorus removal Sludges as revealed by polyphosphate kinase genes. *Applied And Environmental Microbiology*. **73**, 5865-5874 (2007).

Dionisi, H.M. *et al*. Quantification of Nitrosomonas oligotropha-like ammonia-oxidizing bacteria and Nitrospira spp. from full-scale wastewater treatment plants by competitive PCR. *Applied And Environmental Microbiology*. **68**, 245-253 (2002).

Throback, I.N., Enwall, K., Jarvis, Å. & Hallin, S. Reassessing PCR primers targeting nirS, nirK and nosZ genes for community surveys of denitrifying bacteria with DGGE. Fems *Microbiology Ecology*. **49**, 401-417 (2004).

Yan, T.F. *et al*. Molecular diversity and characterization of nitrite reductase gene fragments (nirK and nirS) from nitrate- and uranium-contaminated groundwater. *Environmental Microbiology*. **5**, 13-24 (2003).

Bru, D., Sarr, A. & Philippot, L. Relative abundances of proteobacterial membrane-bound and periplasmic nitrate reductases in selected environments. *Applied And Environmental Microbiology*. **73**, 5971-5974 (2007).

Zhi, W. & Ji, G. Quantitative response relationships between nitrogen transformation rates and nitrogen functional genes in a tidal flow constructed wetland under C/N ratio constraints*.* *Water Research.* **64**, 32-41 (2014).

**Table S2**: Bacterial taxonomic assignment at the phylum level with Illumina MiSeq sequencing. Relative abundance of a bacterial genus was set as the number of sequences affiliated to that bacterial genus divided by the total number of sequences per sample (>2‰).

|  | **R_20_-7** | **R_30_-7** | **R_50_-7** | **R_20_-15** | **R_30_-15** | **R_50_-15** | **R_20_-35** | **R_30_-35** | **R_50_-35** | **R_20_-75** | **R_30_-75** | **R_50_-75** | **R_20_-125** | **R_30_-125** | **R_50_-125** |
| --- | --- | --- | --- | --- | --- | --- | --- | --- | --- | --- | --- | --- | --- | --- | --- |
| **Chlorobi** | 1.65% | 1.75% | 1.92% | 0.59% | 1.17% | 1.02% | 0.68% | 1.04% | 0.58% | 0.46% | 0.24% | 0.31% | 0.49% | 0.10% | 0.27% |
| **Proteobacteria** | 22.92% | 15.48% | 14.28% | 28.09% | 33.79% | 33.21% | 25.95% | 24.67% | 36.27% | 26.04% | 22.06% | 27.46% | 25.12% | 24.70% | 39.98% |
| **Actinobacteria** | 6.61% | 8.69% | 9.08% | 10.84% | 5.04% | 3.22% | 9.10% | 13.97% | 3.73% | 11.08% | 28.98% | 19.95% | 11.04% | 23.35% | 4.80% |
| **Firmicutes** | 3.54% | 1.19% | 1.28% | 2.06% | 1.16% | 1.68% | 2.14% | 1.47% | 2.40% | 1.03% | 1.00% | 1.37% | 1.33% | 0.62% | 0.87% |
| **Bacteroidetes** | 8.21% | 7.18% | 5.97% | 8.28% | 8.27% | 9.92% | 9.55% | 6.45% | 8.68% | 12.01% | 3.49% | 4.94% | 11.54% | 9.86% | 8.68% |
| **TM7** | 0.76% | 0.64% | 0.77% | 0.60% | 0.18% | 0.28% | 0.75% | 0.33% | 0.21% | 1.34% | 0.31% | 0.24% | 1.35% | 0.29% | 0.26% |
| **Chloroflexi** | 2.85% | 5.45% | 5.90% | 2.25% | 0.64% | 0.72% | 2.98% | 1.40% | 1.03% | 2.35% | 0.52% | 1.35% | 2.65% | 0.39% | 1.10% |
| **Acidobacteria** | 2.02% | 1.95% | 1.80% | 1.49% | 1.36% | 1.30% | 1.23% | 1.87% | 1.60% | 0.83% | 0.61% | 0.84% | 1.06% | 0.33% | 0.85% |
| **Nitrospira** | 0.37% | 0.47% | 0.24% | 0.23% | 1.20% | 0.94% | 0.23% | 1.23% | 1.08% | 0.21% | 0.60% | 0.95% | 0.40% | 0.24% | 0.89% |
| **Chlamydiae** | 0.24% | 0.33% | 0.37% | 0.12% | 0.28% | 0.46% | 0.20% | 0.30% | 0.35% | 0.09% | 0.25% | 0.19% | 0.09% | 0.14% | 0.25% |
| **OD1** | 0.10% | 0.39% | 0.23% | 0.04% | 0.18% | 0.40% | 0.12% | 0.11% | 0.07% | 0.05% | 0.07% | 0.07% | 0.06% | 0.07% | 0.18% |
| **Verrucomicrobia** | 0.49% | 1.11% | 0.90% | 0.41% | 0.50% | 0.46% | 0.41% | 0.70% | 0.33% | 0.53% | 0.28% | 0.37% | 0.90% | 0.12% | 0.39% |
| **Planctomycetes** | 0.49% | 0.73% | 0.71% | 0.24% | 0.24% | 0.14% | 0.71% | 0.29% | 0.09% | 0.20% | 0.24% | 0.12% | 0.41% | 0.10% | 0.09% |
| **Armatimonadetes** | 0.24% | 0.15% | 0.11% | 0.04% | 0.02% | 0.03% | 0.11% | 0.01% | 0.01% | 0.04% | 0.01% | 0.01% | 0.05% | 0.01% | 0.04% |
| **unclassfied** | 49.38% | 54.12% | 56.23% | 44.57% | 45.69% | 45.98% | 45.70% | 45.77% | 43.14% | 43.62% | 41.21% | 41.50% | 43.20% | 39.55% | 41.15% |

***The number after dash represents sampling day.**

**Table S3**: Bacterial taxonomic assignment at the genus level with Illumina MiSeq sequencing. Relative abundance of a bacterial genus was set as the number of sequences affiliated to that bacterial genus divided by the total number of sequences per sample (>2‰).

|  | **R_20_-7** | **R_30_-7** | **R_50_-7** | **R_20_-15** | **R_30_-15** | **R_50_-15** | **R_20_-35** | **R_30_-35** | **R_50_-35** | **R_20_-75** | **R_30_-75** | **R_50_-75** | **R_20_-125** | **R_30_-125** | **R_50_-125** |
| --- | --- | --- | --- | --- | --- | --- | --- | --- | --- | --- | --- | --- | --- | --- | --- |
| ***Ignavibacterium*** | 1.16% | 1.24% | 1.36% | 0.42% | 0.82% | 0.72% | 0.48% | 0.73% | 0.41% | 0.32% | 0.17% | 0.22% | 0.35% | 0.07% | 0.19% |
| ***Thermomonas*** | 0.28% | 0.04% | 0.02% | 0.46% | 0.01% | 0.02% | 0.50% | 0.03% | 0.07% | 0.48% | ND | 0.04% | 0.34% | 0.01% | 0.02% |
| ***Pseudomonas*** | 0.64% | 0.34% | 0.12% | 2.45% | 10.92% | 10.89% | 2.04% | 5.30% | 5.72% | 1.97% | 2.11% | 1.52% | 2.06% | 1.66% | 1.08% |
| ***Raoultella*** | 0.60% | ND | ND | 0.78% | 0.01% | 0.01% | 0.74% | ND | 0.03% | 0.28% | ND | ND | 0.29% | ND | ND |
| ***Zoogloea*** | 0.04% | 0.04% | 0.03% | 0.40% | 0.10% | 0.15% | 0.28% | 0.13% | 0.25% | 1.44% | 0.08% | 0.10% | 1.06% | 0.02% | 0.13% |
| ***Albidiferax*** | 0.01% | 0.01% | 0.01% | 0.01% | 0.03% | 0.04% | ND | 0.14% | 0.30% | 0.06% | 0.15% | 0.24% | 0.02% | 0.93% | 0.73% |
| ***Curvibacter*** | ND | ND | ND | ND | ND | ND | 0.01% | 0.06% | 0.16% | ND | 0.06% | 0.23% | ND | 0.50% | 0.60% |
| ***Janthinobacterium*** | 3.42% | 0.11% | 0.05% | 2.53% | 4.01% | 2.79% | 2.31% | 2.75% | 1.31% | 3.65% | 1.80% | 1.41% | 2.98% | 4.51% | 0.69% |
| ***Aquabacterium*** | 0.01% | ND | 0.03% | 0.01% | ND | 0.02% | ND | 0.02% | 1.55% | ND | 0.03% | 0.02% | ND | 0.01% | 0.03% |
| ***Ilumatobacter*** | 0.70% | 1.07% | 1.13% | 1.06% | 0.14% | 0.12% | 0.79% | 0.21% | 0.10% | 0.41% | 0.04% | 0.07% | 0.68% | ND | 0.06% |
| ***Arthrobacter*** | 0.40% | 0.08% | 0.06% | 0.99% | 0.84% | 0.30% | 0.71% | 6.07% | 0.89% | 2.40% | 13.09% | 10.06% | 2.32% | 12.66% | 1.76% |
| ***Clostridium XI*** | 0.24% | 0.16% | 0.20% | 0.17% | 0.18% | 0.32% | 0.16% | 0.30% | 0.56% | 0.06% | 0.23% | 0.31% | 0.13% | 0.12% | 0.19% |
| ***Lactococcus*** | 1.22% | ND | 0.01% | 0.66% | ND | ND | 0.72% | ND | ND | 0.29% | ND | ND | 0.38% | ND | 0.01% |
| ***Chryseobacterium*** | 0.24% | 0.01% | ND | 0.25% | 0.06% | 0.06% | 0.21% | 0.16% | 0.45% | 0.23% | 0.48% | 0.49% | 0.33% | 0.93% | 0.56% |
| ***Flavobacterium*** | 1.39% | 0.13% | 0.08% | 2.77% | 2.05% | 2.22% | 3.02% | 1.37% | 2.23% | 4.24% | 0.69% | 1.05% | 3.68% | 4.20% | 2.62% |
| ***TM7_genera_incertae_sedis*** | 0.54% | 0.45% | 0.54% | 0.42% | 0.13% | 0.19% | 0.53% | 0.23% | 0.15% | 0.95% | 0.22% | 0.17% | 0.95% | 0.21% | 0.19% |
| ***Caldilinea*** | 0.61% | 1.23% | 1.33% | 0.78% | 0.19% | 0.17% | 0.82% | 0.18% | 0.14% | 0.48% | 0.11% | 0.11% | 0.50% | 0.06% | 0.05% |
| ***Gp4*** | 0.73% | 0.45% | 0.35% | 0.45% | 0.13% | 0.18% | 0.37% | 0.14% | 0.17% | 0.32% | 0.07% | 0.14% | 0.35% | 0.06% | 0.13% |
| ***Nitrospira*** | 0.26% | 0.33% | 0.17% | 0.16% | 0.85% | 0.66% | 0.16% | 0.87% | 0.76% | 0.15% | 0.42% | 0.67% | 0.28% | 0.17% | 0.63% |
| ***Serratia*** | 0.01% | 0.01% | ND | 0.01% | 0.05% | 0.02% | ND | 0.22% | 0.24% | ND | 2.64% | 5.14% | ND | 1.84% | 8.42% |
| ***Rhodanobacter*** | ND | ND | ND | 0.04% | ND | ND | 0.01% | 0.03% | 0.01% | 0.02% | 0.25% | 0.18% | 0.04% | 0.65% | 0.07% |
| ***Pedobacter*** | 0.02% | ND | 0.01% | 0.09% | 0.06% | 0.04% | 0.19% | 0.04% | 0.14% | 0.31% | 0.16% | 0.17% | 0.36% | 1.00% | 0.34% |

***ND represents “none detected”, and the number after dash represents sampling day.**

**Table S4**. The whole PLFA profiles (%) of the microorganism in R_20_, R_30_ and R_50_ at 5 °C.

|  | **R_20_-15** | **R_30_-15** | **R_50_-15** | **R_20_-35** | **R_30_-35** | **R_50_-35** | **R_20_-75** | **R_30_-75** | **R_50_-75** | **R_20_-125** | **R_30_-125** | **R_50_-125** |
| --- | --- | --- | --- | --- | --- | --- | --- | --- | --- | --- | --- | --- |
| **9:00** | 0.28 | 1.02 | 0.21 | 0.28 | 0.94 | 0.22 | 0.33 | 0.18 | 0.16 | 0.33 | 0.18 | 0.14 |
| **10:00** | ND | ND | ND | ND | ND | ND | ND | ND | 0.02 | ND | 0.04 | ND |
| **11:0 iso 3OH** | 0.24 | 0.94 | 0.18 | ND | ND | ND | 0.24 | ND | ND | 0.24 | 0.22 | ND |
| **12:0 3OH** | ND | ND | ND | 0.09 | ND | ND | ND | 0.05 | ND | ND | 0.09 | ND |
| **12:1 3OH** | **ND** | **ND** | **ND** | **ND** | **ND** | **ND** | **0.15** | **ND** | **ND** | **ND** | **ND** | **ND** |
| **12:1** | **ND** | **ND** | **ND** | **ND** | **ND** | **ND** | **ND** | **ND** | **ND** | **ND** | **0.03** | **ND** |
| **13:0 iso** | ND | ND | ND | 0.07 | ND | ND | ND | 0.08 | ND | ND | 0.06 | ND |
| **13:0 iso 3OH** | ND | 0.2 | ND | ND | ND | ND | ND | ND | 0.04 | ND | ND | 0.02 |
| **14:0 iso** | 0.96 | 0.89 | 0.2 | 0.73 | 0.78 | 0.11 | 0.49 | 0.48 | ND | 0.42 | 0.50 | ND |
| **14:1 TRANS 9** | **0.77** | **ND** | **ND** | **ND** | **1.09** | **ND** | **0.38** | **ND** | **ND** | **ND** | **0.33** | **0.13** |
| **14:00** | 0.98 | 1.31 | 0.48 | 0.91 | 1.15 | 0.47 | 0.85 | 0.82 | 0.57 | 0.85 | 0.74 | 0.54 |
| **14:0 2OH** | ND | ND | 0.05 | ND | ND | 0.05 | 0.05 | 0.08 | 0.19 | ND | 0.04 | 0.2 |
| **14:1 TRANS 9** | **ND** | **ND** | **0.21** | **0.33** | **ND** | **0.24** | **ND** | **0.37** | **0.14** | **ND** | **ND** | **ND** |
| **15:1 iso G** | **0.66** | **0.7** | **ND** | **0.76** | **0.76** | **ND** | **0.41** | **0.38** | **0.05** | **0.41** | **0.35** | **0.07** |
| **15:1 anteiso** | **1.06** | **0.96** | **ND** | **1.15** | **1.11** | **ND** | **0.43** | **0.64** | **0.09** | **0.41** | **0.62** | **0.08** |
| **15:0 iso** | 1.32 | 0.49 | ND | 1.31 | ND | ND | 0.55 | 0.96 | 0.07 | 0.53 | 0.92 | 0.06 |
| **15:0 anteiso** | 4.87 | 4.14 | 0.25 | 4.84 | 4.11 | 0.28 | 1.9 | 5.71 | 0.47 | 1.82 | 5.4 | 0.42 |
| **15:1** | **ND** | **ND** | **ND** | **ND** | **0.69** | **ND** | **ND** | **0.19** | **ND** | **ND** | **ND** | **0.03** |
| **15:00** | 0.57 | 0.38 | 0.29 | 0.54 | 0.28 | 0.32 | 0.29 | 0.63 | 0.53 | 0.32 | 0.55 | 0.51 |
| **15:0 iso 3OH** | ND | ND | ND | ND | ND | 0.08 | ND | 0.13 | ND | ND | 0.19 | ND |
| **15:0 iso 2OH** | ND | ND | ND | ND | ND | `ND | ND | ND | 0.34 | ND | ND | 0.33 |
| **16:0 iso** | 1.76 | 1.11 | ND | 1.83 | 1.29 | ND | 0.73 | 1.43 | 0.16 | 0.77 | 1.4 | 0.17 |
| **16:0 anteiso** | 0.48 | ND | 0.05 | 0.46 | 0.51 | ND | ND | 0.48 | 0.05 | ND | 0.41 | 0.06 |
| **(Table S4, Continued)** |  |  |  |  |  |  |  |  |  |  |  |  |
| **16:1 cis 9** | **13.94** | **21.82** | **4.83** | **14.06** | **21.6** | **4.97** | **19.49** | **29.67** | **8.94** | **19.49** | **28.35** | **8.93** |
| **16:1** | **2.24** | **1.14** | **ND** | **2.26** | **1.03** | **ND** | **0.45** | **2.00** | **0.08** | **0.43** | **2.04** | **ND** |
| **16:00** | 32.64 | 27.71 | 32.59 | 32.66 | 27.56 | 32.97 | 36.6 | 26.05 | 39.42 | 36.67 | 24.94 | 39.3 |
| **16:0 10 methyl** | ND | 0.86 | ND | ND | 0.87 | ND | 0.28 | ND | ND | 0.95 | ND | 0.03 |
| **16:0 iso 2OH** | 0.16 | ND | ND | ND | ND | ND | ND | ND | ND | ND | ND | ND |
| **16:0 iso 3OH** | 0.22 | ND | ND | 0.25 | ND | ND | ND | 0.41 | ND | ND | 0.38 | ND |
| **16:0 2OH** | ND | 0.52 | 0.58 | 0.07 | 0.36 | 0.56 | ND | 0.29 | 0.76 | ND | 0.24 | 0.76 |
| **16:0 3OH** | ND | ND | ND | ND | ND | ND | 0.48 | 0.15 | ND | 0.44 | 0.12 | ND |
| **16:0 9 methyl** | 0.42 | ND | ND | 0.33 | ND | ND | ND | 0.49 | ND | ND | 0.75 | ND |
| **16:1 iso** | **ND** | **ND** | **ND** | **ND** | **0.26** | **ND** | **0.1** | **0.21** | **0.03** | **ND** | **0.18** | **0.03** |
| **17:1 anteiso** | **0.15** | **ND** | **ND** | **0.11** | **ND** | **ND** | **0.16** | **0.1** | **ND** | **ND** | **0.15** | **ND** |
| **17:0 iso** | 0.14 | ND | ND | ND | ND | ND | ND | 0.07 | ND | ND | 0.07 | ND |
| **17:0 anteiso** | 1.51 | 0.72 | 0.15 | 1.45 | 0.74 | 0.14 | 0.58 | 0.99 | 0.18 | 0.92 | 0.93 | 0.19 |
| **17:1 cis 9** | **0.47** | **0.56** | **0.3** | **0.44** | **0.52** | **0.31** | **0.72** | **0.26** | **0.52** | **0.93** | **0.22** | **0.54** |
| **17:00** | 0.29 | ND | 0.24 | 0.31 | ND | 0.18 | 0.78 | 0.23 | 0.2 | ND | 0.22 | 0.21 |
| **17:0 iso 3OH** | 0.14 | ND | ND | 0.11 | 0.56 | ND | 0.24 | 0.29 | 0.07 | 0.26 | 0.24 | 0.1 |
| **17:0 2OH** | ND | ND | ND | ND | ND | ND | ND | 0.39 | ND | 0.11 | 0.41 | ND |
| **17:0 anteiso 2OH** | 0.29 | ND | ND | ND | ND | ND | ND | ND | ND | ND | ND | ND |
| **17:1 iso** | **ND** | **ND** | **ND** | **ND** | **ND** | **ND** | **0.06** | **ND** | **ND** | **ND** | **ND** | **ND** |
| **17:0 cyclo** | ND | ND | ND | 0.26 | ND | ND | ND | 0.29 | ND | ND | 0.28 | ND |
| **18:3 cis 6,12,14** | **ND** | **0.39** | **0.06** | **ND** | **0.34** | **0.08** | **ND** | **0.13** | **0.05** | **ND** | **ND** | **0.05** |
| **18:2 cis 9,12** | **2.1** | **8.86** | **15.62** | **2.18** | **8.7** | **15.83** | **1.26** | **5.66** | **26.22** | **1.28** | **5.5** | **26.31** |
| **18:1 cis 9** | **2.33** | **8.56** | **25.67** | **2.24** | **8.61** | **25.83** | **1.29** | **ND** | **ND** | **1.27** | **4.33** | **ND** |
| **18:00** | 19.46 | 12.96 | 16.99 | 19.81 | 12.71 | 17.14 | 24.75 | 13.51 | 19.78 | 24.93 | 12.94 | 19.88 |
| **18:0 3OH** | 0.17 | ND | ND | 0.11 | ND | ND | ND | ND | ND | ND | ND | ND |
| **(Table S4, Continued)** |  |  |  |  |  |  |  |  |  |  |  |  |
| **18:1 cis 11/t 9/t 6** | **7.06** | **ND** | **ND** | **7.22** | **ND** | **ND** | **4.1** | **4.69** | **ND** | **4.21** | **4.21** | **ND** |
| **18:0 2OH** | ND | ND | ND | ND | ND | ND | ND | ND | 0.05 | ND | ND | 0.05 |
| **19:00** | ND | 0.09 | ND | ND | ND | ND | ND | ND | ND | ND | ND | ND |
| **19:0 anteiso** | 0.12 | ND | ND | ND | ND | ND | ND | ND | ND | ND | ND | ND |
| **20:1 cis 11** | **ND** | **ND** | **ND** | **ND** | **ND** | **ND** | **ND** | **ND** | **0.14** | **ND** | **ND** | **0.13** |
| **20:00** | 0.53 | ND | 0.23 | ND | ND | 0.19 | 0.37 | 0.15 | 0.2 | 0.31 | 0.18 | 0.21 |
| **20:4 cis 5,8,11,14** | **ND** | **ND** | **ND** | **0.44** | **ND** | **ND** | **ND** | **0.16** | **0.04** | **0.14** | **0.18** | **0.04** |

***ND represents “none detected”, the number after dash represents sampling day, and** **the UFA values are in bold Time New Roman.**

**
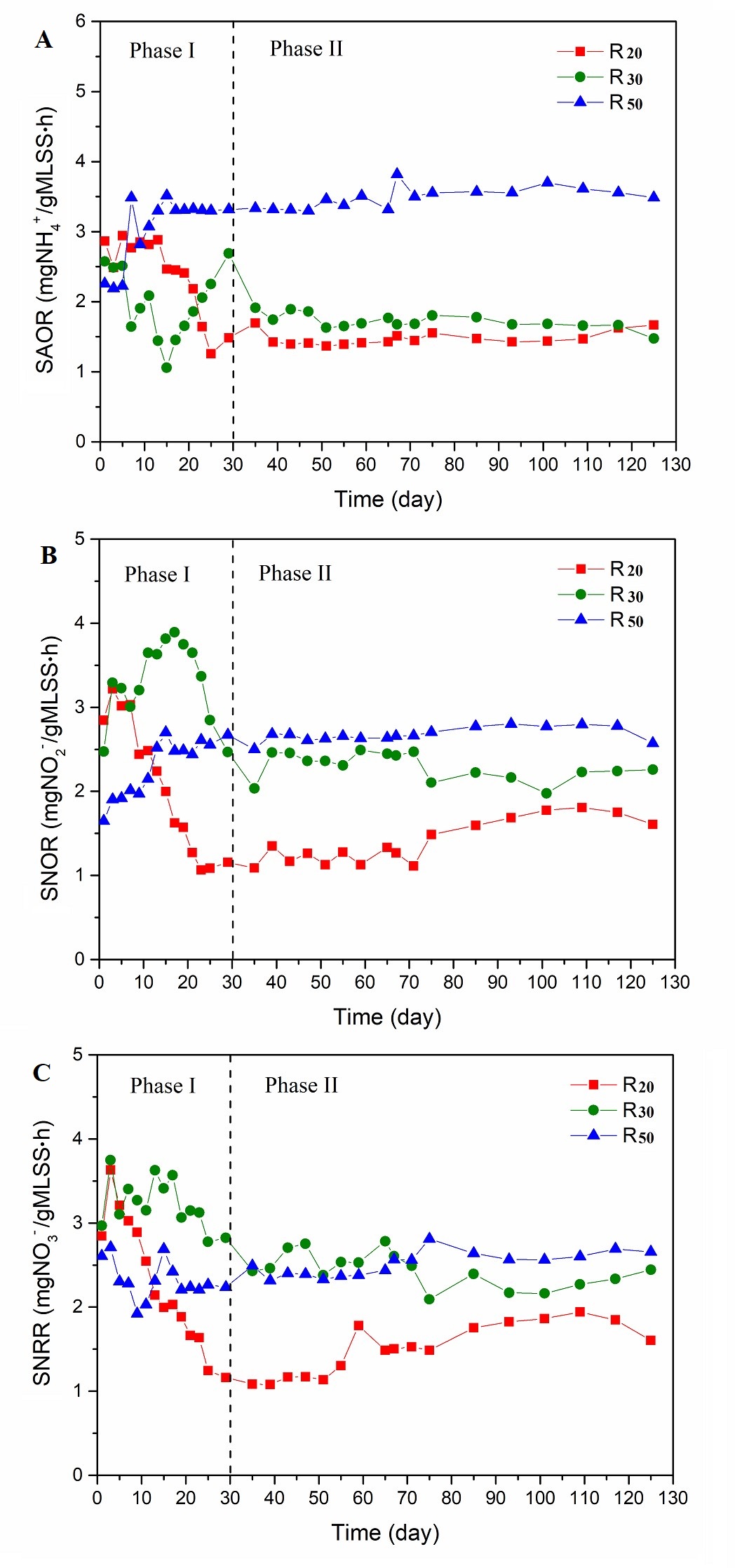
**

**Fig. S1** Variations of SAOR (A), SNOR (B), and SNRR (C) of R_20_, R_30_ and R_50_ at 5 °C.


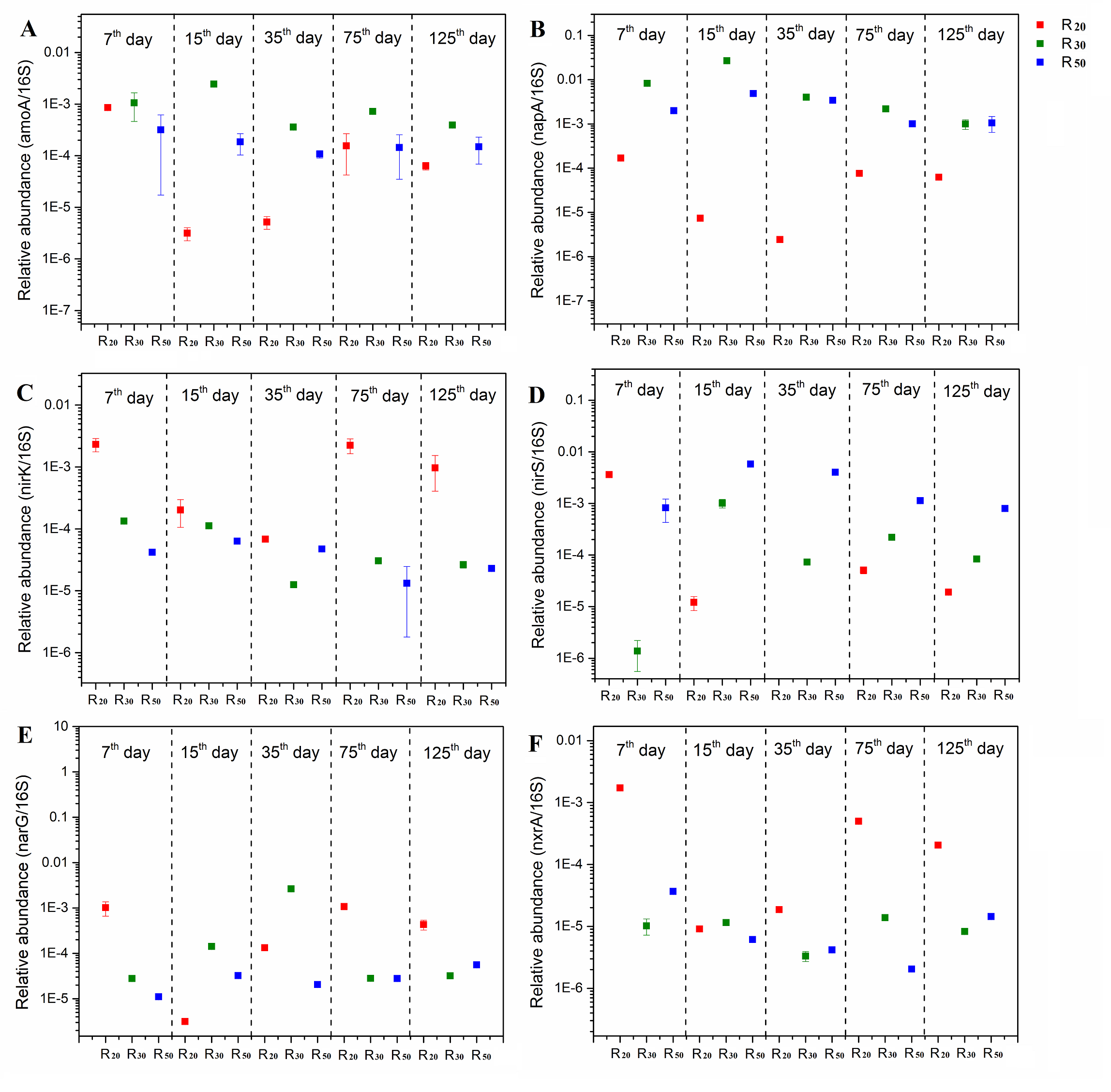


**Fig. S2** Relative functional genes abundance of amoA (A), napA (B), nirK (C), nirS (D), nirG (E) and nxrA (F) during different periods of R_20_, R_30_ and R_50_ at 5 °C.

**
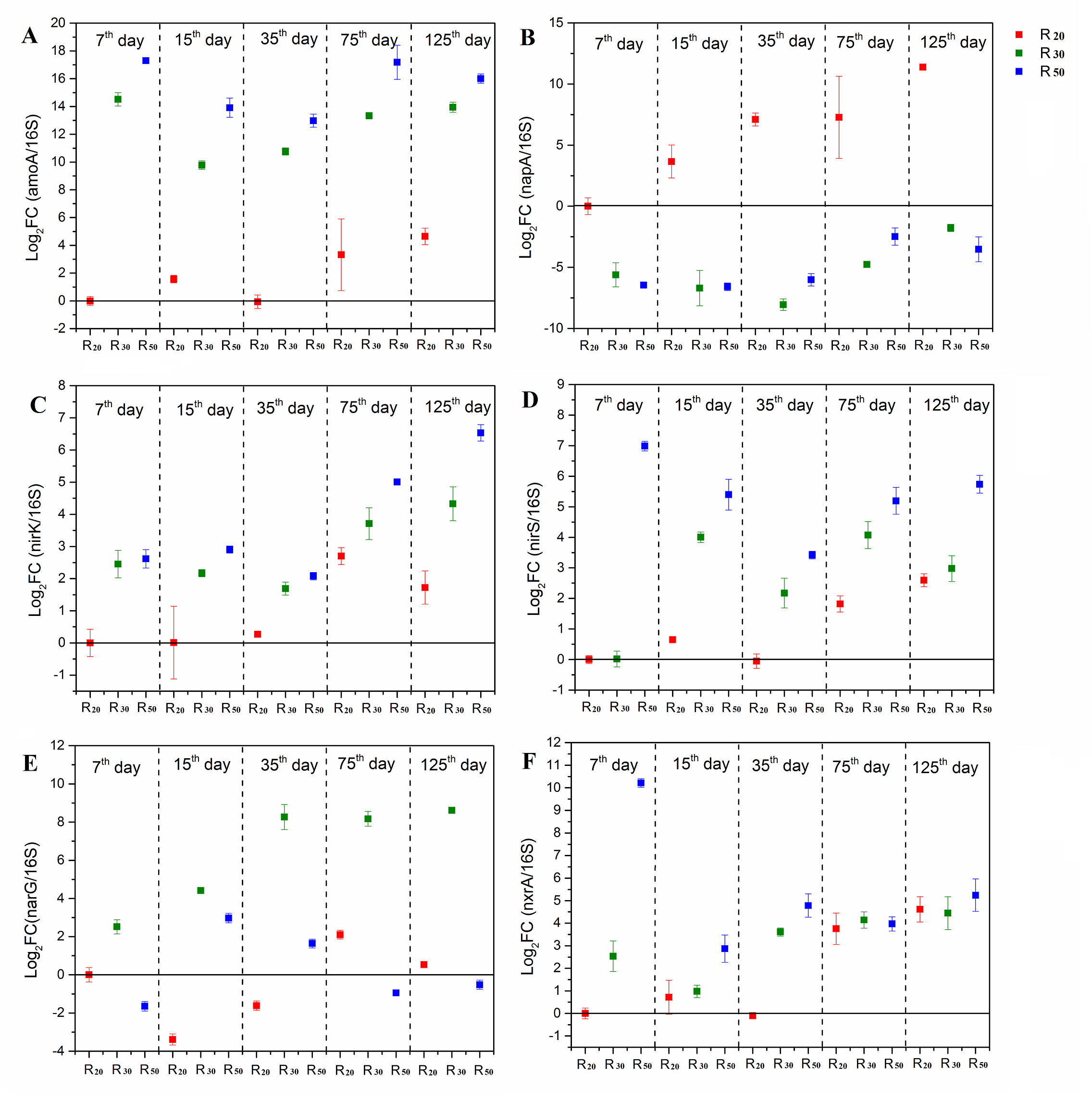
**

**Fig. S3** Fold changes in amoA (A), napA (B), nirK (C), nirS (D), nirG (E) and nxrA (F) expression as compared to R4-7^th^ day sample during different periods of R_20_, R_30_ and R_50_ at 5 °C.

**
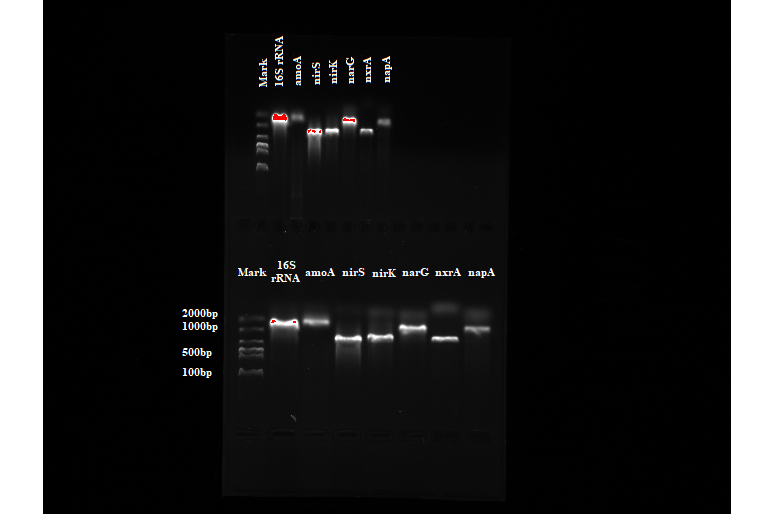
**

**Fig. S4** PCR amplification functional gene in the seeding sludge. Marker, DNA Marker DL 2000.


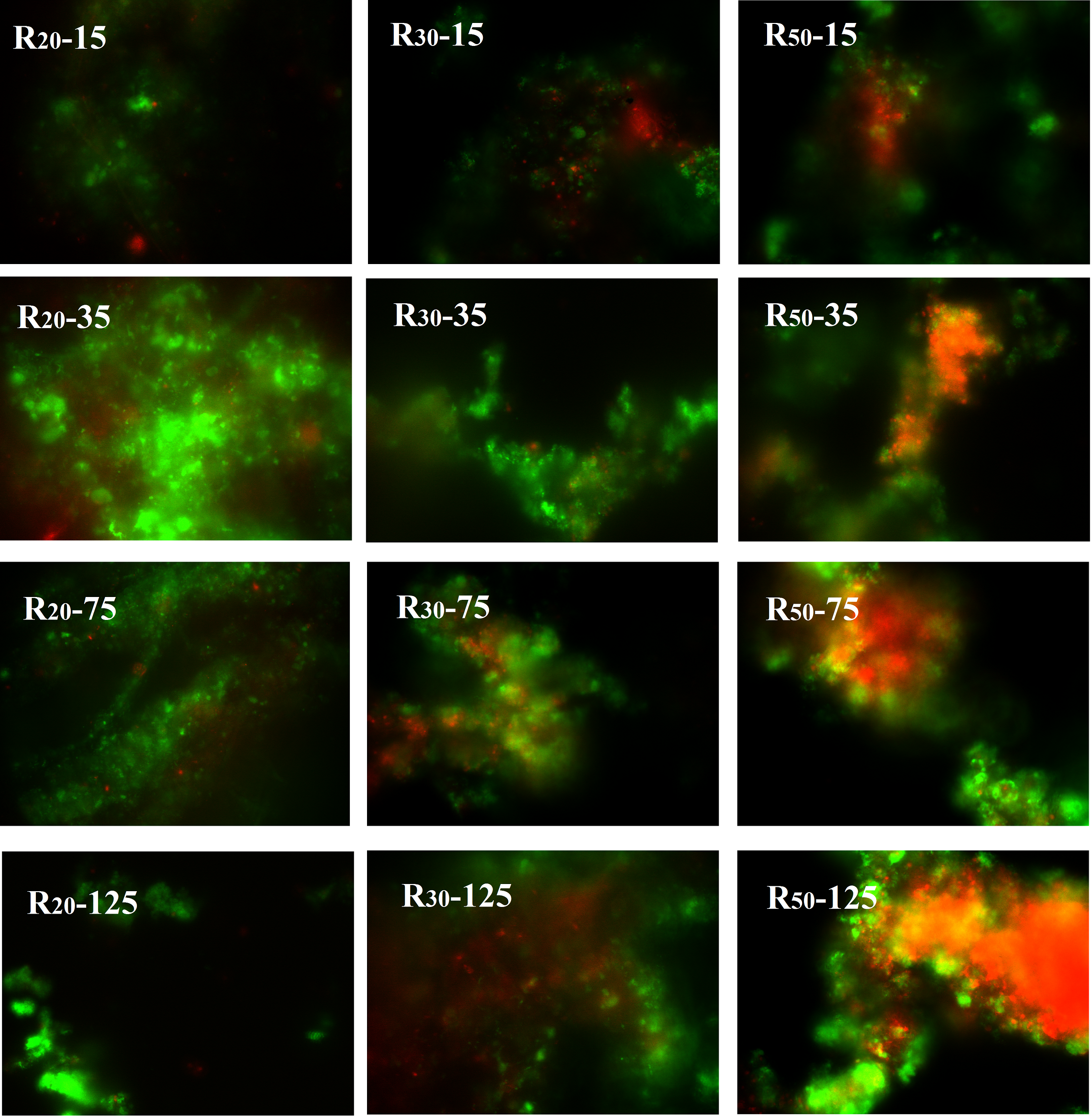


**Fig. S5** Fluorescence microscopic images in R_20_, R_30_ and R_50_ with different sampling times. The samples were viewed under × 100 magnification. Live cells are stained green, dead cells are stained red. The number after dash represents the sampling day.
